# Cardiac Standstill With Intracardiac Clot Formation

**DOI:** 10.5811/cpcem.2019.7.42932

**Published:** 2019-09-30

**Authors:** Jeffrey Tadashi Sakamoto, Ian Storch, Laleh Gharahbaghian

**Affiliations:** * Stanford/Kaiser Emergency Medicine Residency Program, Department of Emergency Medicine, Palo Alto, California; † University of Florida College of Medicine Jacksonville, Department of Emergency Medicine, Jacksonville, Florida; ‡ Stanford University School of Medicine, Department of Emergency Medicine, Palo Alto, California

## Abstract

This case describes and depicts cardiac standstill with thrombosed blood within the chambers of the heart. This was likely due to stasis of blood from a prolonged no-flow state. After viewing this ultrasound finding, the decision was made to halt resuscitative efforts in this case of a patient in cardiac arrest.

## CASE PRESENTATION

**Image 1:**
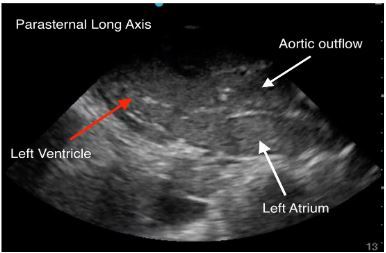
Transthoracic echocardiography parasternal long view of the heart demonstrating echogenic (thrombosed) blood within the left atria, left ventricle, and aortic outflow.

**Image 2:**
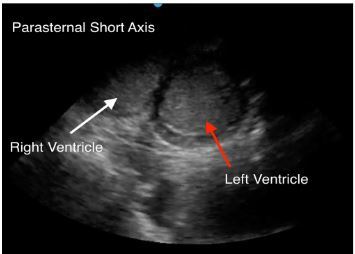
Transthoracic echocardiography: parasternal short view of the heart demonstrating echogenic (thrombosed) blood within the left and right ventricles.

An 88-year-old female with gastric cancer presented to the emergency department (ED) in cardiac arrest. She was at an outpatient clinic when she lost pulses. Cardiopulmonary resuscitation (CPR) was initiated by emergency medical services upon their arrival after a downtime of several minutes without chest compressions. Upon arrival in the ED, CPR was in progress. During a rhythm and pulse check, transthoracic echocardiography was performed demonstrating cardiac standstill, as well as a collection of echogenic material within the ventricles. This finding represented thrombosed blood (Image 1–2 and Video). After visualizing cardiac standstill with intracardiac clots, the decision was made to stop resuscitation.

## DISCUSSION

Cardiac standstill is defined as the lack of movement of the valves and free wall of the heart. When visualized on ultrasound during cardiac arrest assessment it has been discussed as a potential endpoint to CPR. Cohort studies have shown that cardiac standstill was associated with a very low rate of survival from 0–0.6%.^1^,^2^ In contrast, cardiac activity on ultrasound during cardiac arrest is strongly associated with return of spontaneous circulation and survival, and therefore provides prognostic information that may guide resuscitation efforts.^3^

This case also demonstrates clot formation within the heart, which is hypothesized to be from stasis of blood after a prolonged downtime prior to initiating CPR. Due to the initial lack of chest compressions, it is possible that there was a period without blood flow through the heart causing coagulation. This patient’s history of cancer also may have contributed to a hypercoagulable state. There are no case reports discussing intracardiac clots in cardiac arrest, but this type of echocardiography pattern has been described to be fine, speckled, and with a uniform appearance.^4^ Perhaps with further investigation, this echocardiography pattern could be used to identify cardiac arrest patients with a prolonged downtime and potentially shed light on a prognosis if extensive intracardiac clots were found with or without ongoing cardiac activity.

**CPC-EM Capsule**

What do we already know about this clinical entity?

*Increased clot burden can be caused by a low-flow state, create an even lower flow state, and can increase strain on the myocardium*.

What is the major impact of the image(s)?

*Rarely do you see this amount of clot burden inside both ventricles of a patient with a witnessed arrest and cardiopulmonary resuscitation. It provides a clear illustration*.

How might this improve emergency medicine practice?

*The presence of significant clot burden in both ventricles could give the physician an indication of prolonged downtime*.

## Supplementary Information

Video. Transthoracic echocardiography: parasternal long view of the heart demonstrating cardiac standstill and thrombosed blood within the heart chambers.
